# Machine Learning-Enabled Rapid Assessment of Plant-Based Protein Digestibility Through Physicochemical Profiles

**DOI:** 10.3390/foods14223874

**Published:** 2025-11-13

**Authors:** Meichen Liu, Ruoyan Zhang, Hao Yin, Yu Zhong, Yapeng Fang, Cuixia Sun, Yun Deng

**Affiliations:** Department of Food Science and Technology, Shanghai Jiao Tong University, 800 Dongchuan Road, Shanghai 200240, China; hzau_lmc3@163.com (M.L.); zhangruoyan0629@sjtu.edu.cn (R.Z.); 4399hhh@sjtu.edu.cn (H.Y.); zhangruoyan2021@126.com (Y.Z.); zhangruoyancau@gmail.com (Y.F.); 4399lmc@sina.com (C.S.)

**Keywords:** artificial intelligence, feedforward neural network, plant-based protein, digestibility, physicochemical indicators

## Abstract

Plant-based proteins offer sustainable alternatives to animal sources, yet their lower digestibility remains a critical barrier to widespread applications. Current digestibility assessment methods require days of analysis and gram-scale samples, creating significant bottlenecks in protein optimization workflows. This study developed an ensembled deep learning framework that transforms digestibility prediction from a resource-intensive process to a rapid, minimal-sample assessment. By systematically characterizing 23 diverse plant protein isolates across multiple physicochemical dimensions, we trained a feedforward neural network based on augmented data. Our model identified α-helix content, random coil content, and solubility as key digestibility indicators. This insight enabled the construction of a streamlined three-feature model that reduced assessment time by 80% while requiring only one-hundredth of standard sample amounts. When validated against independent published datasets, the model achieved rational prediction accuracy, with an R^2^ = 0.91. These findings establish a transformative framework for accelerating plant protein development, enabling rapid screening of novel sources and targeted modification strategies to enhance nutritional bioavailability, ultimately advancing sustainable food system transitions.

## 1. Introduction

Plant-based proteins have gained significant global popularity. The primary dietary sources of plant proteins include beans, grains, seeds, and nuts, while pseudocereals and algae account for a relatively smaller proportion of total intake [1]. Compared to animal-based diets, they offer numerous benefits, including human health promotion, environmental friendliness, resource sustainability, and animal welfare improvement. Increasing daily intake of plant-based food can reduce the risk of chronic diseases brought by cholesterol, and it is a solution to lactose intolerance [2], offering plant-based diets a promising future. Further research and development of plant-based protein products should be guided by scientific formulations and innovative processing techniques, given the growing public health concerns related to obesity and shifts in diet. In 2020, the global plant-based protein market was valued at USD 29.4 billion, and it is expected to reach USD 162 billion by 2030 [3]. Further research and development of plant-based protein products should be encouraged by scientific formulations and innovative processing techniques, as obesity has become a public health concern with changes in diet and lifestyle.

However, the low digestibility of plant-based proteins limits their wide application in human food. In general, the digestibility is in the range of 45% to 80%, while that of animal proteins can exceed 90% [4]. Food digestion can be divided into three main stages, the oral, gastric, and intestinal phases, while protein is mainly digested in the gastric and intestinal phases [5]. The final hydrolyzed products are small peptides and free amino acids, which can be absorbed by the human body [6]. The bioaccessibility depends on the physicochemical properties of the protein, like particle size, zeta potential, solubility, intermolecular forces, and secondary structures. It has been reported that protein in a soluble state is more likely to be broken down by digestive enzymes [7]. In addition, an increase in protein digestibility is often accompanied by a reduction in particle size and an increase in the zeta potential absolute value. In intermolecular forces, disulfide bonds play a dominant role in protein aggregation and deaggregation. The bond drives protein molecule folding, making it more difficult for digestive enzymes to bind with the specific sites, thus reducing digestibility. Therefore, breaking the formation of disulfide bonds can improve the digestibility of proteins [8]. The α-helix and β-sheet, maintained by hydrogen bonds, are regular structures in the secondary structures, while the β-turn and random coil belong to irregular structures. Generally, protein digestibility decreases as the content of the β-sheet increases. In plant proteins, the β-sheet content can account for 44.0%, while in animal proteins, it only accounts for 7.0% to 11.0%, which explains why plant-based proteins are less digestible [9]. It is necessary to understand the protein digestibility for a better application of plant-based protein resources.

The static digestion model primarily simulates the physiological conditions of food digestion in the oral, gastric, and small intestinal phases. The INFOGEST model is currently an internationally standardized and widely adopted in vitro static digestion model. It regulates the types and concentrations of digestive enzymes, environmental pH and temperature, digestion time, and salt concentration, which addresses the issue of data incomparability caused by differences between models [10]. The conventional digestibility assay is a labor-intensive and time-consuming procedure, involving lengthy preparation of digestive solutions and substantial manual effort for nitrogen quantification via the Kjeldahl method, which is universally utilized to assess the protein content [8,11]. It typically requires gram-level quantities of protein samples, while the assessment of solubility and secondary structure can be performed with only milligram-level amounts, making them more suitable for limited or precious sample conditions.

These limitations restrict their rapid or high-throughput applications. Hence, more recently, researchers have focused on replacing the protein content directly with easily measurable indicators like spectral, which can be determined effortlessly. Machine learning (ML) is an extensive field that enables computers to make smart decisions [12]. The feedforward neural network (FNN), a branch of ML, refers to a network design that uses the error back-propagation algorithm to construct a model. An FNN model consists of input layers, hidden layers, and output layers. Through multiple layers of linear weighting and non-linear transformation, the network automatically learns the mapping between input data and output results. It has the advantages of convenient data input and a stable system [13]. The FNN has been widely applied in food quality control, ingredient analysis, adulteration detection, and shelf-life prediction [12]. Nowadays, integrating spectral technology with an FNN model to detect protein content has become a promising trend [14,15,16]. However, the input spectral data consist of high-dimensional wavelength signals that require dimensionality reduction, and spectral information is easily affected by factors such as sample condition. The physicochemical indicators used as inputs are a small set of well-defined parameters that are relatively stable and highly reproducible. An insufficient number of studies have concentrated on deciphering the relationship between the physicochemical properties of the protein and its digestibility by machine learning models, highlighting a current research gap.

Hence, more recently, researchers have focused on replacing the protein content directly with other properties that can be determined effortlessly. Machine learning (ML) is an extensive field that enables computers to make smart decisions [12]. A feedforward neural network (FNN), a branch of ML, refers to a network design that uses the error back-propagation algorithm to construct a model. An FNN model consists of input layers, hidden layers, and output layers. Through multiple layers of linear weighting and non-linear transformation, the network automatically learns the mapping between input data and output results. It has the advantages of convenient data input and a stable system [13]. The FNN has been widely applied in food quality control, ingredient analysis, adulteration detection, and shelf-life prediction [12]. Nowadays, integrating spectral technology with FNN models to detect protein content has become a promising trend [14,15,16]. However, the input spectral data consist of high-dimensional wavelength signals that require dimensionality reduction, and spectral information is easily affected by factors such as sample condition. The physicochemical indicators used as inputs are a small set of well-defined parameters that are relatively stable and highly reproducible. An insufficient number of studies concentrated on deciphering the relationship between the physicochemical properties of the protein and its digestibility by machine learning models.

Despite growing recognition of physicochemical properties’ influence on protein digestibility, no systematic framework exists to leverage these relationships for rapid digestibility prediction. Standard approaches require days of analysis and gram quantities of samples, while emerging spectroscopic methods lack the mechanistic insights needed for targeted protein modification. Machine learning offers unprecedented opportunities to decode complex structure–function relationships, yet its application to plant protein digestibility remains unexplored. We hypothesize that specific physicochemical signatures form a predictive fingerprint of digestibility that can be captured through deep learning algorithms. Therefore, this study aims to (1) comprehensively characterize the physicochemical profiles of 23 diverse plant protein isolates; (2) develop and validate an ensemble deep learning framework, including data augmentation and prediction, for digestibility; (3) identify the minimal set of features required for accurate assessment; and (4) establish a rapid screening protocol that accelerates sustainable protein development. By bridging fundamental protein science with artificial intelligence, our work promises to transform how we discover, optimize, and deploy plant-based proteins for human nutrition.

## 2. Materials and Methods

### 2.1. Materials

Soybean protein isolate 1 (SPI1), pea protein isolate 1 (PPI1), fava bean protein isolate 1 (FPI1), kidney bean protein isolate 1 (KPI1), chickpea protein isolate 1 (CPI1), lupin protein isolate 1 (LPI1), rice protein isolate 1 (RPI1), brown rice protein isolate 1 (BPI1), wheat protein isolate 1 (WhPI1), barley protein isolate 1 (BaPI1), oat protein isolate 1 (OPI1), corn protein isolate 1 (CoPI1), potato protein isolate 1 (PoPI1), walnut protein isolate 1 (WPI1), and sesame protein isolate 1 (SePI1) were obtained from Xian Xuquan Biotechnology Co., Ltd. China (Xi’an, China). They were dried by the spray-drying technique.

Soybean protein isolate 2 (SPI2), pea protein isolate 2 (PPI2), fava bean protein isolate 2 (FPI2), chickpea protein isolate 2 (CPI2), rice protein isolate 2 (RPI2), oat protein isolate 2 (OPI2), walnut protein isolate 2 (WPI2), and sesame2 protein isolate 2 (SePI2) were prepared in the laboratory.

The protein content of the above materials is presented in Table S1. All of the chemical reagents in this study were of analytical grade and bought from Sinopharm Group (Shanghai, China).

### 2.2. Methods

#### 2.2.1. Preparation of Protein Isolates

Protein isolates were extracted according to Zhang et al. [17] with slight modifications. Soybean2, pea2, fava bean2, chickpea2, rice2, oat2, walnut2, and sesame2 experienced peeling and drying, and then they were ground into powders. The dried powder was mixed with n-hexane at a ratio of 1:20 (*w*/*v*) and subjected to an ultrasonic process at room temperature for 2 h to remove fat. The defatted plant powder was combined with distilled water at a ratio of 1:20 (*w*/*v*), and the pH was adjusted to 10.0 by adding 1 mol/L NaOH, followed by stirring for 1 h. The mixture was centrifuged at 3000 r/min for 15 min, and the supernatant was collected. The pH of the supernatant was adjusted to 4.0 by adding 1 mol/L HCl and was then left overnight. Afterwards, the mixture was centrifuged at 3000 r/min for 15 min to obtain the precipitate, which was washed three times with distilled water. The washed precipitate was then freeze-dried to obtain plant-based protein isolate, which was stored in a cool and dry place for future determination.

#### 2.2.2. Measurement of Protein Content

The protein content of the plant-based protein isolate was determined using the Kjeldahl method in accordance with the method described in GB 50095-2016 [18]. The plant-based protein isolate (0.19 g to 0.21 g) was weighed into a Kjeldahl’s digestive tube, followed by the addition of 3.50 g of K_2_SO_4_, 0.40 g of CuSO_4_·5H_2_O, and 12.50 mL of 1.84 g/L H_2_SO_4_ for digestion in a digestion furnace. The digestion product was then transferred to a semi-automatic Kjeldahl apparatus, where 50 mL of 40% NaOH (*w*/*v*) was added and then distilled. The released ammonia was absorbed using 30 mL of 40 g/L H_3_BO_3_ solution. Finally, the sample was titrated with 0.1022 mol/L hydrochloric acid solution with two drops of 0.1% bromocresol green (*w*/*v*) and 0.1% methyl red (*w*/*v*) as an indicator. The data obtained was expressed as % nitrogen content and then converted to crude protein by multiplying the nitrogen content by a factor of 6.25.

(1) $$PC\;(\%)=\frac{V\times c\times 0.014}{m}\times 6.25\times 100\%$$

where *PC* is the protein content (%), *V* is the volume of the hydrochloric acid solution consumed (mL), *c* is the concentration of the hydrochloric acid solution (mol/L), and *m* is the weight of the plant-based protein isolate (g).

#### 2.2.3. Particle Size and Zeta Potential Measurement

The method of Zhang [19] was followed. The 1 mg/mL plant-based protein isolate solution was prepared by a 0.01 mol/L phosphate buffer (pH 7.0). The solution was centrifuged at 3000 r/min for 15 min to collect the supernatant. The particle size and zeta potential were recorded by a nanoparticle size and zeta potential analyzer (NanoBrook Omni, New York, NY, USA) with distilled water as the dispersant at 25 °C at a 40 mW laser.

#### 2.2.4. Solubility

A procedure based on the work of Dong et al. [20] was used to identify protein solubility. The plant-based protein isolate solution was diluted to a concentration of 10 mg/mL and subsequently extracted at room temperature for 1 h. The mixture was then centrifuged at 10,000 r/min for 10 min. The supernatant was collected for further analysis. Solubility was calculated as the protein content in the supernatant using the Coomassie Brilliant Blue method.

#### 2.2.5. Identification of Intermolecular Interactions

Firstly, five denaturing agents were prepared to alter different intermolecular interactions. Solution A was made up of 0.05 mol/L NaCl. Solution B was made up of 0.6 mol/L NaCl. Solution C was made up of 0.6 mol/L NaCl and 1.5 mol/L urea. Solution D was composed of 0.6 mol/L NaCl and 8 mol/L urea. Solution E included 0.6 mol/L NaCl, 8.0 mol/L urea, and 1.0 mol/L β-mercaptoethanol. The above solutions were all dissolved in a 0.01 M phosphate buffer (pH 7.0). A total of 0.5 g of plant-based protein isolate was homogenized with 5 mL of the five solutions above, respectively, followed by centrifugation at 3000 r/min for 20 min. The supernatant was collected, and the protein content was determined using the Bradford method (Sangon Biotech Co., Ltd., Shanghai, China) using bovine serum albumin (BSA) as the standard [21]. Protein content was measured using a Multiskan SkyHigh multifunctional enzyme marker (Thermo Fisher Scientific, Waltham, MA, USA). Differences in the protein content dissolved in the extraction solvents were used to calculate the content of different bonds, with units expressed in mg/L. SB-SA denoted the content of ionic bonds. SC-SB denoted the content of hydrogen bonds. SD-SC denoted the content of hydrophobic interactions, and SE-SD denoted the content of the disulfide bond [22]. SA, SB, SC, SD, and SE refer to the content of protein in Solution A, Solution B, Solution C, Solution D, and Solution E.

#### 2.2.6. Secondary Structure Analysis

Circular dichroism spectra (CDS) were obtained from a J-1500 spectrometer (JASCO, Tokyo, Japan). The plant-based protein isolate was dissolved in a 0.01 mol/L phosphate buffer (pH 7.0) to 100 μg/mL. The solution was centrifuged at 3000 r/min for 15 min, and the supernatant was collected for further inspection. A 1 cm path-length quartz cuvette was used, and data were recorded from 190 to 250 nm with deionized water as the background at 25 °C. The sensitivity was set as 200 mdeg/cm, and the resolution was 0.1 nm. The results were recorded as the mean of five scans. The relative percentage of the α-helix, β-sheet, β-turn, and random coil contents was estimated by Circular Dichroism Neural Network software (SELCON3) (version 2021) [23].

#### 2.2.7. In Vitro Digestion with INFOGEST 2.0

In vitro digestion was performed using the INFOGEST 2.0 static model with adjustments [24,25]. For the gastric phase, 1.0 g of plant-based protein isolate powder was dissolved in 9 mL of distilled water. The solution was mixed with 10 mL of simulated gastric fluid (SGF), which contained 9 mL of SGF stock solution, 10 μL of 0.3 mol/L CaCl_2_(H_2_O)_2_, and 80 mg of pepsin. The pH was adjusted to 3.5 using 1 mol/L HCl, ensuring a final pepsin activity of 2000 U/mL. Then, the mixture was shaken at 37 °C for 2 h, followed by the inactivation of pepsin by adjusting the pH by adding 7.0 with 1 mol/L NaOH. In the intestinal digestion, 20 mL of simulated intestinal fluid (SIF) was added to the gastric digestion product. SIF was composed of 19 mL of an SIF stock solution, 80 μL of 0.3 M CaCl_2_(H_2_O)_2_, 2.8 mg of trypsin, 5.0 mg of chymotrypsin, and 128 mg of bile salts. The trypsin activity was 100 U/mL. The mixture was also shaken at 37 °C for 2 h. The reaction was terminated by a boiling water bath for 10 min.

The final gastric and intestinal digestion products were mixed with an equal volume of a 10% trichloroacetic acid (TCA) (*w*/*v*) solution. The mixture was incubated for 30 min and then centrifuged at 12,000 r/min for 15 min at 4 °C. The precipitate was dried at 50 °C for further protein content analysis. The digestibility was calculated by the following formula:

(2) $$Digestibility\;\left(\%\right)\;=\;\left(1-\frac{PC_{1}}{PC_{0}}\right)\times 100\%$$

where *PC*_1_ denotes the protein content in the dried precipitate after digestion and *PC*_0_ represents the initial protein content in the protein isolate before digestion.

### 2.3. Machine Learning

#### 2.3.1. Data Preprocessing

Firstly, the physicochemical profile of 23 samples was standardized using StandardScaler before data augmentation and building the feedforward neural network (FNN) model, and it was calculated by Equation (3) as follows:

(3) $$Y_{s}=\frac{Y_{i}-Y_{m}}{Y_{std}}$$

where *Y_s_* is the standardized data, *Y_i_* is the original data, *Y_m_* is the mean value of each property, and *Y_std_* represents the standard deviation.

#### 2.3.2. Data Augmentation

Deep learning models, including variational autoencoders (VAEs), generative adversarial networks (GANs), mixed sample data augmentation (Mixup), and K-nearest neighbors (KNNs), were employed to learn data patterns from 23 experimental samples. VAEs learned the latent variable distribution and generated 1% of the targeted new data. The data synthesized by GANs accounted for 1% as well. Mixup improved model robustness, contributing to 98% of the final data. KNNs supplemented the rest of the data, ensuring a smoother distribution. Finally, the data was expanded 20-fold, reaching 483 samples, consistent with the original data’s dimensional and distributional characteristics. This enriched dataset was prepared for further construction of an FNN model to predict digestibility.

#### 2.3.3. FNN Model Construction and Training

An FNN model was constructed using the torch package in Python (version 2023.2.1). It was trained on the basis of 483 pieces of augmented samples to explore the relationship between protein characteristics and digestibility. The training set constituted 80% of the data, and the test set constituted 20% of the data. StandardScaler was applied for the data standardization according to Equation (3).

The FNN architecture consisted of four fully connected layers with ReLU activation, dropout regularization, and progressive feature reduction to enhance generalization and mitigate overfitting. The model was trained for 300 epochs using a batch size of 16, with the mean square error (MSE) as the loss function. An early stopping strategy was adopted when no improvement was observed for 15 consecutive epochs.

#### 2.3.4. FNN Model Evaluation

Model evaluation was based on the mean absolute error (MAE), MSE, RMSE, and R^2^ calculated by Equations (4)–(7).

(4) $$MAE=\frac{1}{N}\sum_{i=1}^{N}\left|\left.Y_{test}-Y_{pre}\right|\right.$$

(5) $$\mathrm{MSE}=\frac{1}{N}\sum_{i=1}^{N}{\left(Y_{test}-Y_{pre}\right)}^{2}$$

(6) $$\mathrm{RMSE}=\sqrt{\frac{1}{N}\sum_{i=1}^{N}{\left(Y_{test}-Y_{pre}\right)}^{2}}$$

(7) $$R^{2}=1-\frac{\sum_{i=1}^{n}{\left(Y_{test}-Y_{pre}\right)}^{2}}{\sum_{i=1}^{n}{\left(Y_{test}-\bar{Y_{test}}\right)}^{2}}$$

where *Y_test_* represents the real digestibility of the samples and *Y_pre_* is the predicted digestibility of the samples.

### 2.4. Statistical Data Analysis

All experiments were conducted with at least three replicates, and data are presented as mean ± standard deviation. The significance analysis is provided in Table S2. Data analysis and figure plotting were carried out using GraphPad Prism 8.0 software. Data augmentation and construction of the FNN model were performed in PyCharm Community Edition 2023.2.1, and relevant figures were generated.

## 3. Results and Discussion

### 3.1. Analysis of Physicochemical Properties of Different Types of Plant-Based Proteins

#### 3.1.1. Particle Size and Zeta Potential

**Figure 1:**
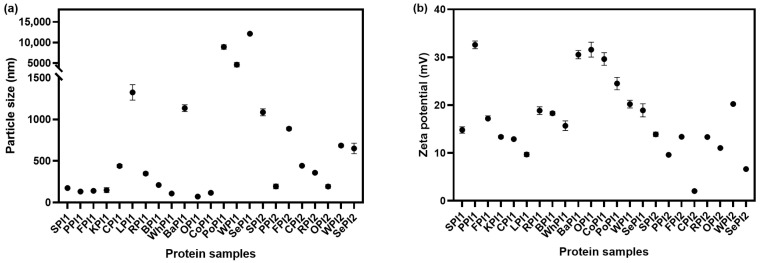
Particle size (**a**) and zeta potential (**b**) of different kinds of plant-based protein isolates.

It was observed that average particle sizes were lower than 1500 nm, except for PoPI1, WPI1, and SePI1. The results indicated that proteins derived from tubers and nuts were in larger aggregates than those from beans and grains (Figure 1). Smaller particle size improves the digestibility of the protein by enhancing interaction with digestive enzymes [26]. The particle size reflects the degree of dispersion in aqueous solution. The particle sizes of nut proteins extracted by the freeze-drying technique, like walnut and sesame, were 685.55 nm and 651.47 nm, respectively, which decreased compared with the proteins made by spray-drying. However, the protein sizes of soybean, pea, fava bean, chickpea, rice, and oat were larger when they were prepared by the freeze-drying technique. This result suggested that freeze-drying had a significant impact on breaking down the nut proteins. It is attributed to the fact that spray-drying caused the re-aggregation of ruptured proteins [27,28], which might do harm to the digestibility.

As shown in Figure 1b, the absolute value of the zeta potential ranged from 2.05 mV to 25.61 mV, which reflected the electrostatic repulsion between particles. It has been recognized that the absolute value of the zeta potential of a stable protein dispersion system is over 25 mV. PPI1, BaPI1, OPI1, and CoPI1 remained relatively steady, while the absolute value of the zeta potential of CPI2 and SePI2 was only 2.05 mV and 6.64 mV. The larger the absolute value of the zeta potential, the more stable the system; conversely, lower zeta potential values reduce stability, potentially leading to aggregation and flocculation in the solution [29]. Plant proteins showed distinct sensitivity to processing methods due to their subunit composition [30]. Especially, the zeta potential absolute value of pea protein isolate dropped from 32.61 mV to 9.61 mV in spray- and freeze-drying. It was obvious that spray-drying contributed to stabilizing the protein systems when compared to the same plant proteins made from two drying techniques. The protein structure could be stretched by the proper drying method, and the inside amino acid residues were exposed, thus raising the zeta potential absolute value.

#### 3.1.2. Solubility

**Figure 2:**
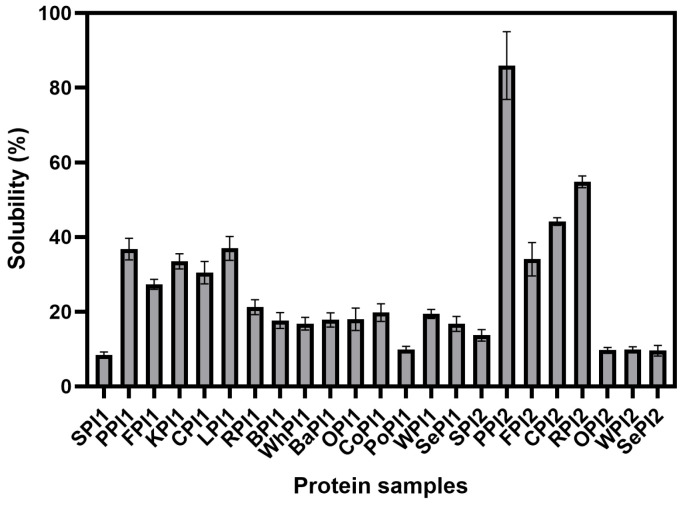
Solubility of different kinds of plant-based protein isolates.

In this study, the solubility of plant-based proteins ranged from 8.41% to 85.94% (Figure 2). The covalent and non-covalent interactions of proteins affected their solubility. The low solubility of PoPI1 was mainly due to protein denaturation during extraction and purification, which leads to increased surface hydrophobicity [31]. Comparing the proteins extracted from beans, soybean protein was less soluble, and pea protein showed higher solubility. We could observe that the solubility of proteins from different origins had distinct sensitivities to the preparation method. Bean proteins were more soluble when made by freeze-drying, while grain and nut proteins were more likely to dissolve in water with the spray-drying technique. Bean proteins undergo partial denaturation during spray-drying, resulting in reduced solubility. In contrast, spray-drying causes partial unfolding or swelling of grain and nut proteins, increasing their water-accessible surface and making them more readily soluble [32]. It has been proven that interactions between protein and the structure can affect solubility. Protein aggregation had a negative effect on solubility, which limits enzyme access [33]. The increase in protein solubility drives the increase in its digestibility. This is because the protein in the soluble state is more sensitive to digestive enzymes, which creates more chances to break down [7]. It is important to increase the solubility of plant proteins through extracting and processing methods, as they have a lower digestibility rate naturally [33].

#### 3.1.3. Intermolecular Forces

Protein structure is partly upheld by ionic bonds, hydrogen bonds, hydrophobic interactions, and disulfide bonds, thus altering the protein digestibility [34]. To further figure out the relationship between the conformation and the digestibility rate, we determined the intermolecular forces. Five denaturing solutions, containing varying concentrations of NaCl, urea, and β-mercaptoethanol, were used to disrupt different intermolecular interactions in the proteins. For each solution, 0.5 g of plant-based protein isolate was homogenized and centrifuged, and the supernatant was collected. Protein content was measured using the Bradford assay. By comparing the amount of protein solubilized in each solution, the contributions of ionic bonds, hydrogen bonds, hydrophobic interactions, and disulfide bonds were estimated [22].

**Figure 3:**
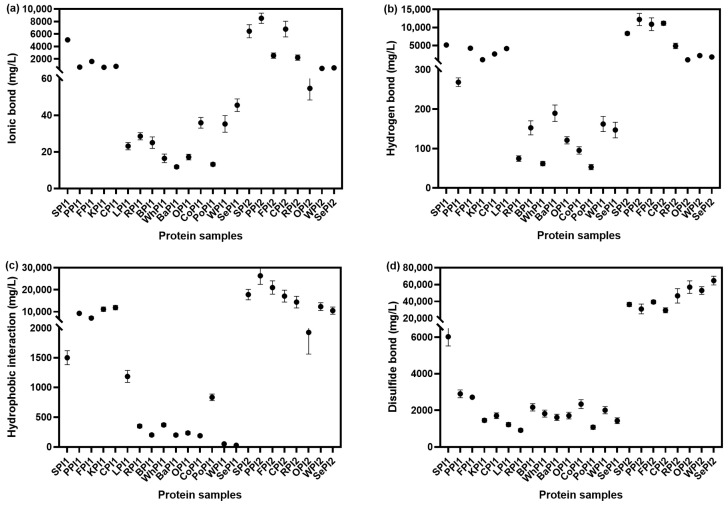
Intermolecular forces of different kinds of plant-based protein isolates. Ionic bond (**a**), hydrogen bond (**b**), hydrophobic interaction (**c**), and disulfide bond (**d**).

Figure 3a displayed that ionic bonds are more abundant in proteins made by freeze-drying. The force is an electrostatic interaction between oppositely charged amino acid residues, which maintains the three-dimensional network structure of proteins [35]. Hydrogen bonds help the maintenance of secondary structures, like the α-helix, β-sheet, β-turn, and random coil. The break and formation occur in the initial phase of enzyme digestion. From Figure 3b, we could observe that proteins from bean sources contained more hydrogen bonds than grain and nut sources. PPI had the most abundant hydrogen bonds, and its digestibility was the highest as well. Previous research revealed that hydrophobic interactions took the dominant position of the intermolecular forces in mediating protein structure. More than 50% alteration can be accounted for by hydrophobic interactions [36]. In our study, the content of hydrophobic interactions showed similar change patterns to those of hydrogen bonds (Figure 3c). Disulfide bonds could fold the protein and stabilize the tertiary and quaternary structures, making the protein resistant to enzyme degradation [37]. Apparently, there were more disulfide bonds in proteins dried by freezing than in those made by spraying. The disulfide bonds in freeze-drying proteins ranged from 29,525.2 mg/L to 64,736.9 mg/L (Figure 3d), while the disulfide bonds in spray-drying proteins were less than 6025 mg/L. An increase in disulfide bond content promotes a more compact protein structure, leading to intermolecular aggregation and, consequently, reduced protein digestibility [38].

#### 3.1.4. Secondary Structure

**Figure 4:**
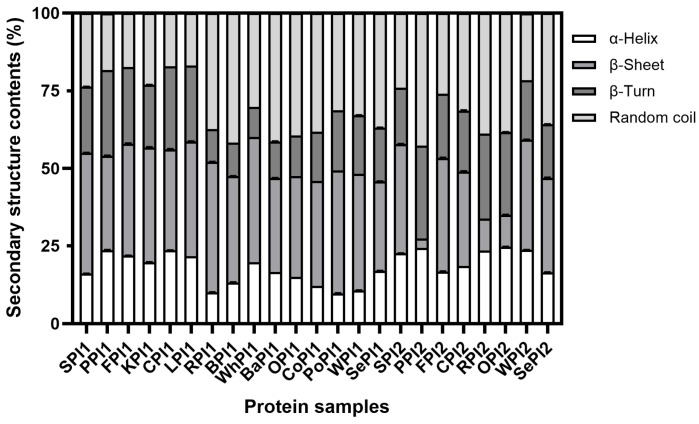
The relative percentage of the secondary structure of different kinds of plant-based protein isolates.

**Figure 5:**
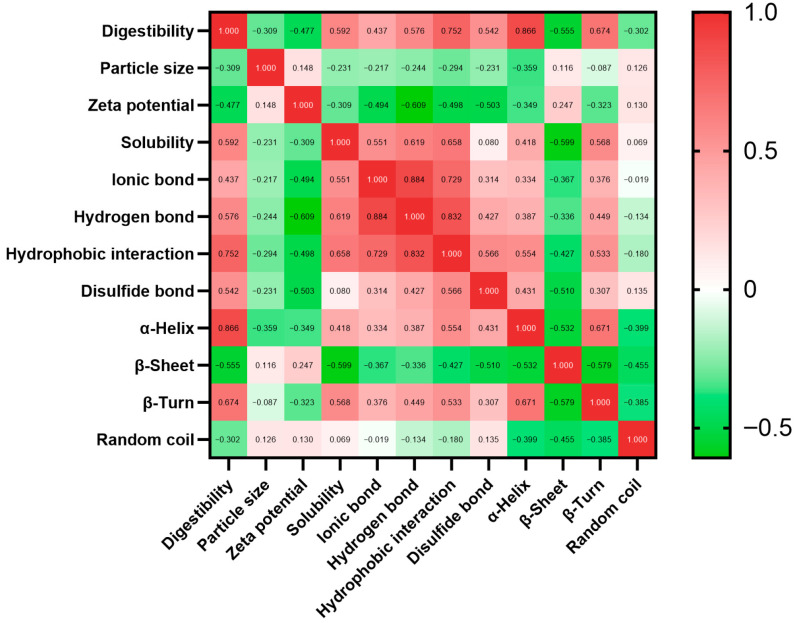
The Pearson analysis between the physicochemical properties (particle size, zeta potential, solubility, intermolecular forces, and secondary structure) and digestibility.

The α-helix, β-sheet, β-turn, and random coil constitute the secondary structure of the protein, which fills in the gap between the amino acid sequence and the three-dimensional spatial structure [39]. Figuring out the secondary structure modifications is essential for protein digestibility prediction, as they are the driving causes of molecular folding and polymerization [40]. From Figure 4, the β-sheet was the dominant secondary structure in all samples, which could make up 42% of the composition. The β-sheet, along with the α-helix, is a regular secondary structure, but it is less likely to be degraded by digestive enzymes compared to the random coil and β-turn [41]. This is consistent with our finding that the β-sheet was negatively correlated with digestibility (Figure 5). We found that regular secondary structures in PPI2 only took up 27.2%, and PPI2 had the highest digestive rate (88.51%) as well. The Pearson correlation coefficient between the β-turn, a non-regular secondary structure, and digestibility was 0.674 (Figure 5), which indicated that elevating the account of the non-regular secondary structure could improve protein digestibility. The β-turn introduced flexibility and compacted folding in the protein backbone, which alleviated spatial constraints between adjacent residues, facilitating enzyme access and potentially enhancing digestibility [40,42].

### 3.2. Analysis of In Vitro Digestibility of Different Types of Plant-Based Proteins

Conducting in vivo studies using animals or humans to investigate food digestion and absorption characteristics has certain limitations, including long experimental periods, high costs, technical complexity, poor reproducibility, and significant ethical concerns. Therefore, there is a need for in vitro models that can closely simulate the physiological processes occurring during digestion. The INFOGEST model simulates food digestion in the oral, gastric, and small intestinal phases, including physiological conditions such as digestive enzymes, pH, temperature, digestion time, and salt concentration. However, since it does not include brush border peptidases, it cannot provide the fully hydrolyzed products of protein sources [10,43].

**Figure 6:**
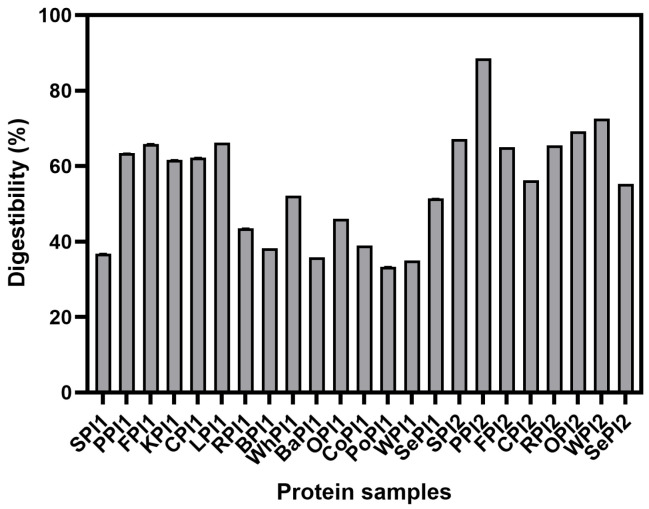
The digestibility of different kinds of plant-based protein isolates.

The digestive rate of PPI2 (88.51%) was the highest, and PoPI2 exhibited the lowest (33.34%). PoPI1, BaPI1, and SePI1 showed relatively low digestibility, suggesting that these proteins might have more compact structures or contain a higher proportion of indigestible bound forms (Figure 6). The proteins made by spray-drying generally displayed lower digestibility than the proteins made by freeze-drying. Proteins are broken down into amino acids and oligopeptides in the gastrointestinal phase [44]. The stomach plays a primary role in digestion, breaking down proteins into small peptides. In the small intestine, pancreatic enzymes continue to degrade proteins, further hydrolyzing peptides, which are ultimately absorbed [45]. This guarantees that proteins can be absorbed by the body and that essential amino acids are able to meet the body’s nutritional needs. The structural feature of the protein is the fundamental cause of the protein’s bioavailability [11]. The high digestibility of PPI2 can be attributed to its outstanding solubility and low content of the β-sheet in the secondary structure. The particle size (8941.79 nm) of PoPI1 was large, and it was less soluble (9.86%) compared to other proteins.

### 3.3. Linear Regression Analysis of the Physicochemical Profile and Digestibility

Multiple linear regressions involving more than one feature provide a linear function to model the relationship between variables [46]. According to a general guideline, the absolute value of the Pearson correlation coefficient (|r|) quantifies the high (|r| ≥ 0.8), moderate (0.8 > |r| ≥ 0.5), or low (0.5 > |r| ≥ 0.3) correlations [47]. Except for the α-helix (|r| = 0.87), there was no high correlation between other physicochemical characteristics and digestibility, though linear regression was efficient in interpretation and computation (Figure 5). As a result, a simple linear function could hardly be found for predicting digestibility using the determined 11 features.

### 3.4. Construction of the Protein Digestibility Prediction Model

#### 3.4.1. Data Augmentation

Due to the failure of building a linear model, a deep learning model for predicting digestibility was taken into account based on the physicochemical profile. To solve the challenge of limited data in constructing a prediction model for protein digestibility, data augmentation was considered, as reported by Zhang [48]. We employed the VAE, GAN, Mixup, and KNN techniques to learn the data patterns of the 23 samples determined from experiments. The details of the data-generating models are presented in Table 1. At first, StandardScaler was used for standardization to make the original data have a mean of 0 and a standard deviation of 1.

The VAE, consisting of an encoder and a decoder, could generate new data points by learning the latent variable distribution of the data [49]. The input was 11 indexes of protein characteristics, and then they were converted into 10 latent dimensions. In the 300 training epochs, real data was compressed into a latent space by the encoder and decoded into reconstructed data. To make the augmented data more like the real data, the parameters of the VAE models were continuously adjusted in the 300 epochs by Adam, which calculated the MSE. The data was adapted to the GAN model to synthesize multi-dimensional data based on the real data we detected [50]. The optimizer and loss function of the GAN model were the same as the VAE to generate new samples that resembled real data. The GAN model had undergone parameter optimization after 300 training iterations. In order to increase the robustness of the synthetic data, we applied Mixup techniques in creating new samples. We picked two samples in the original data randomly and mixed them together by the alpha coefficient in the beta distribution. A total of 450 fake samples were obtained in the Mixup model. The KNN was another data augmentation method employed to supplement a small number of reasonable new data points, which made the data distribution smoother. For each sample we had now, the KNN found its 20 nearest neighbors. The model interpolated randomly between the nearest neighbors, where alpha followed a uniform distribution between 0.1 and 0.9. Finally, the rest absent samples were filled.

All of the augmented data by the VAE, GAN, Mixup, and KNN was stacked into a whole dataset. The four methods had their own strengths. The VAE and GAN were capable of learning data distribution, generating new samples, and enhancing the data diversity. Mixup could improve the robustness of the model by linear mixing, which contributed 98% of the final dataset. The remaining data gaps could be supplemented by the KNN. As such, the original data was scaled by a factor of 20, and we succeeded in acquiring 483 samples. Using the above stacked models, the dimensional and distributional patterns of sufficient new data were the same as the original data detected by experiments.

#### 3.4.2. Training and Validation of the Prediction Model

An artificial neural network is a basic and widely used computational model for processing non-linear data, which emulates the structure and function of the neural network in the human brain [51]. In this research, a feedforward neural network (FNN) model was constructed and trained with the generated new data in order to explore the relationship between the protein characteristics (particle size, zeta potential, solubility, intermolecular forces, and secondary structure) and protein digestibility.

The training set constituted 80% of the data and the test set constituted 20% of the data to ensure the generalization ability of the model. StandardScaler was also used to center the 483 pieces of sample data around the mean and scale each feature to have a unit variance, which could improve the convergence rate of gradient descent and prevent some features from dominating the training process [52].

**Figure 7:**
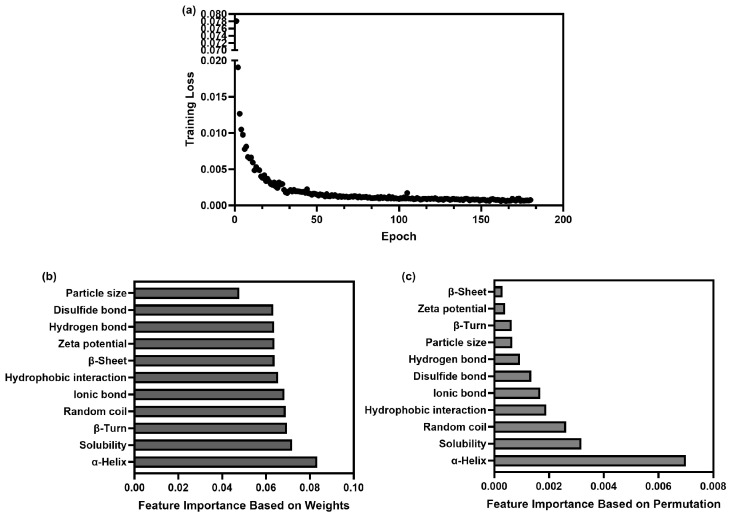
The results of the constructed FNN model for digestibility prediction. The training loss curve of the FNN model (**a**). The feature importance calculated by weights (**b**). The feature importance calculated by permutation (**c**).

The network was composed of four fully connected linear layers, with ReLU activation, dropout regularization, and progressive feature reduction to improve generalization and avoid overfitting (Table 2). The optimized hyperparameters were used to initialize the model, where the training iteration was set at 300 epochs with a batch size of 16. The training criterion function was the MSE. Since the FNN model sought to minimize the objective function via gradient descent, lower loss values indicated more accurate predictions. The early stopping strategy monitored loss and stopped training at 180 epochs, as no improvement had been seen for 15 consecutive epochs, helping the model avoid overfitting and generalize better while reducing computation [53] (Figure 7). Key performance metrics were used to evaluate the predictive accuracy of the model [54]. In the FNN model, the MAE, MSE, RMSE, and R^2^ were 0.014, 0.00051, 0.023, and 0.97, respectively. Low MAE, MSE, and RMSE values and a high R^2^ demonstrated the high predictive accuracy of the FNN model.

The original samples were split into five folds, with each fold serving as the validation set in turn and the remaining four folds used for training. The R^2^ values on each fold ranged from 0.915 to 0.972, with an average of 0.95 ± 0.02; the RMSE and MAE were 0.029 ± 0.005 and 0.020 ± 0.003, respectively, indicating that the model performed stably across different data splits with low variance. In addition, multiple independent training runs with different random seeds showed minimal variation in the results, suggesting that the model is insensitive to initialization (Supplementary Material).

#### 3.4.3. Screening of Potential Characteristics for Predicting Digestibility

The significance of features is essential for clarifying the model and understanding the relationship between the protein characteristics and digestibility. It is more complex when dealing with black box models, like neural networks, than traditional linear models [50]. In this research, we applied weight-based approaches to reveal the relative importance of each protein characteristic.

The weight-based feature importance in the FNN model refers to the absolute values in the first layer, which enhances the interpretability of the model. It can reflect the contribution of a feature variable to the final result [55]. Figure 7b shows that the α-helix (weight = 0.083), solubility (weight = 0.072), and β-turn (weight = 0.070) were the most important features in predicting protein digestibility in the model. The hydrogen bond (weight = 0.064), disulfide bond (weight = 0.063), and particle size (weight = 0.048) had a relatively slight impact on the prediction. We could conclude that it was secondary structure, not intermolecular forces, that played a decisive role in protein digestibility. The feature importance calculated by permutation gave the same results as above. It is another technique for evaluating the significance of a feature in the predictive model. This algorithm tells how much the FNN’s performance deteriorates when some feature is randomly shuffled. The greater the drop in the performance, the more important the feature is [56]. The α-helix (weight = 0.0070), solubility (weight = 0.0032), and random coil (0.0026) took up the first three places in the ranking (Figure 7c), which accounted for 61.90% of the contribution. It can be further deduced that modifying the secondary structure and improving the solubility of the protein might be helpful in increasing its digestibility.

**Figure 8:**
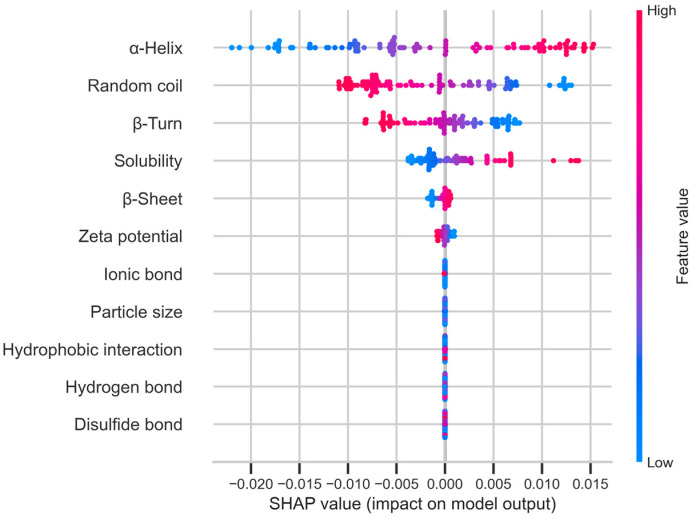
The SHAP summary plot for the FNN model explains the contributions of the physicochemical properties.

The SHAP value was also used to reveal how physicochemical characteristics drove the model’s prediction and highlight their positive or negative contributions. As shown in Figure 8, the α-helix ranked at the top of the SHAP summary plot, indicating that it was the most important feature. Red dots, which represented high α-helix content, occupied the right side with positive SHAP values, suggesting that higher α-helix content tended to increase the predicted digestibility. The random coil also showed a positive contribution to the model, but it had a weaker influence compared to the α-helix.

Since the α-helix, random coil, and solubility are significant, they were chosen to be input into the FNN model to validate its robustness. If the model presented high predictive accuracy for digestibility, then these indicators could be used as indirect predictors of digestibility, thereby reducing the need for time-consuming experimental measurements. In the simplified FNN model with three inputs, the MAE, MSE, RMSE, and R^2^ score were 0.023, 0.00095, 0.030, and 0.93, respectively. The results indicated that digestibility could be accurately predicted by the model using the α-helix, random coil, and solubility. Though the simplified model reduced experimental workload and sample usage, there was a small decrease compared to the full model.

It has been proven that protein aggregation has a negative effect on solubility, which limits enzyme access [33]. The increase in protein solubility drives the increase in its digestibility, which is because protein in a soluble state is more sensitive to digestive enzymes, creating more chances to be broken down [7]. The secondary structure has a great impact on the compactness of protein folding and has a significant impact on the action of digestive enzymes [39]. Hence, these indicators could be measured to estimate digestibility, providing a more efficient and less labor-intensive alternative to direct digestibility assays.

#### 3.4.4. The Application of the Simplified FNN Model with Three Inputs to Estimate Protein Digestibility

To evaluate whether the simplified model was suitable for other plant proteins, we compared the data reported in the literature with the outputs of the model [57,58,59,60,61]. Table 3 presents the real digestibility of different plant protein sources, along with the predictions from our simplified FNN model. In general, the model achieved an accuracy of R^2^ = 0.91, an MAE = 4.74, and an RMSE = 5.82 on an independent validation set. It captured the trends of plant-based proteins’ digestibility well, especially for quinoa and kiwifruit protein. However, these values indicated that the errors were relatively larger for some high-digestibility samples, while the errors were smaller for low-digestibility samples. In addition, solubility had a positive correlation with digestibility, which was the same as the results explained by the SHAP value.

## 4. Conclusions

The study measured the intermolecular forces, secondary structures, and other physicochemical properties of different plant-based proteins and their impact on digestibility. The results proved that particle size, zeta potential, solubility, and intermolecular forces varied significantly among plant protein sources and were influenced by drying methods. Secondary structure analysis revealed that a higher β-sheet content negatively affected digestibility, whereas an increased β-turn fraction was beneficial. The complex relationship between the digestibility and other physicochemical properties indicated that a simple linear regression model is not suitable for predicting the protein digestibility. Hence, we have established a neural network model, an FNN, with 11 features to capture the interactions between protein features and digestibility, with high prediction accuracy (R^2^ = 0.97). The model identified the α-helix content, random coil content, and solubility as the most influential factors in digestibility prediction. However, there still exist some limitations in the present study. The model training relied on synthetic data augmentation to a large extent, which could introduce risks of overfitting or label leakage. The experiments were conducted on protein isolates only, without considering the effects of complex food matrices, weakening applicability to real foods. In the future, the FNN can be used to predict protein digestibility with the above three characteristics instead of the time-consuming method to determine protein digestibility. Integrating the simplified FNN model with AI-guided formulation optimization can accelerate the rational design of next-generation high-digestibility plant proteins, contributing to sustainable nutrition and precision food engineering.

## Figures and Tables

**Table 1:** Data augmentation models, architecture, and configuration.

Model	Hyperparameter	Value
VAE	latent_dim	10
	encoder_layers	3
	decoder_layers	3
	learning_rate	0.001
	loss_function	MSELoss
GAN	latent_dim	10
	learning_rate	0.001
	loss_function	MSELoss
Mixup	alpha	0.3
	num_samples	450
KNN	k	20
	num_samples	2
	distance_metric	c
	alpha_range	(0.1, 0.9)

**Table 2:** The architecture and configuration of the FNN model for protein digestibility prediction.

	Hyperparameter	Value
**Data processing**	test_size	80% train, 20% test
	batch_size	16
	scaler	StandardScaler
**FNN architecture**	input_dim	11
	fc1	Linear (input_dim→128)
	fc2	Linear (128→64)
	fc3	Linear (64→32)
	fc4	Linear (32→1)
	activation	ReLU
	dropout	*p* = 0.3 (applied after fc1 and fc2)
**Training parameters**	num_epochs	300
	learning_rate	0.0005
	optimizer	Adam
	loss_function	MSELoss
	early_stopping_patience	15
**Feature importance**	weight_importance	Mean absolute value of fc1.weight
	permutation_importance	Difference in the MSE after permuting each feature

**Table 3:** The application of the simplified model to the reported data.

Plant Sources	Solubility	α-Helix Content	Random Coil Content	Digestibility	Prediction Results	Percentage Error (%)
Lentil protein	92.7	10.44	17.43	84.9	93.5	10.13
Quinoa protein	82.94	19.99	16.91	81.07	83.58	3.1
	87	17.49	18.24	84.06	82.39	1.99
	88.53	16.74	18.44	85.15	92.07	8.13
Pearl millet protein	60	30.19	25.79	71.73	65.92	8.1
	62.35	21.48	32.8	75.89	69.16	8.87
Kiwifruit protein	16.51	15	33	35	35.28	0.8
	14.54	10	32	44	43.56	1
Sunflower meal protein	64.48	15.54	17.69	93.67	83.97	10.36

## Data Availability

The original contributions presented in this study are included in the article/Supplementary Materials. Further inquiries can be directed to the corresponding author.

## Supplementary Materials

The following supporting information can be downloaded at https://www.mdpi.com/article/10.3390/foods14223874/s1, Table S1. The protein content of the plant materials. Table S2. Significance analysis of 11 indicators.

## References

[1] Kumar M., Tomar M., Punia S., Dhakane-Lad J., Dhumal S., Changan S., Senapathy M., Berwal M.K., Sampathrajan V., Sayed A.A.S. (2022). Plant-based proteins and their multifaceted industrial applications. LWT.

[2] Shi Z., Dun B., Wei Z., Liu C., Tian J., Ren G., Yao Y. (2021). Peptides Released from Extruded Adzuki Bean Protein through Simulated Gastrointestinal Digestion Exhibit Anti-inflammatory Activity. J. Agric. Food Chem..

[3] Grácio M., Oliveira S., Lima A., Boavida Ferreira R. (2023). RuBisCO as a protein source for potential food applications: A review. Food Chem..

[4] van Vliet S., Burd N.A., van Loon L.J.C. (2015). The Skeletal Muscle Anabolic Response to Plant- versus Animal-Based Protein Consumption1. J. Nutr..

[5] Bourlieu C., Ménard O., Bouzerzour K., Mandalari G., Macierzanka A., Mackie A.R., Dupont D. (2014). Specificity of infant digestive conditions: Some clues for developing relevant in vitro models. Crit. Rev. Food Sci. Nutr..

[6] Aghababaei F., McClements D.J., Hadidi M. (2024). Ultrasound processing for enhanced digestibility of plant proteins. Food Hydrocoll..

[7] Davalos-Vazquez A., Mojica L., Sánchez-Velázquez O.A., Castillo-Herrera G., Urías-Silvas J.E., Doyen A., Moreno-Vilet L. (2024). Techno-functional properties and structural characteristics of cricket protein concentrates affected by pre-treatments and ultrafiltration/diafiltration processes. Food Chem..

[8] Yousif N.E., El Tinay A.H. (2001). Effect of fermentation on sorghum protein fractions and in vitro protein digestibility. Plant Foods Hum. Nutr..

[9] Zhang J., Wang J., Li M., Guo S., Lv Y. (2022). Effects of heat treatment on protein molecular structure and in vitro digestion in whole soybeans with different moisture content. Food Res. Int..

[10] Minekus M., Alminger M., Alvito P., Ballance S., Bohn T., Bourlieu C., Carrière F., Boutrou R., Corredig M., Dupont D. (2014). A standardised static in vitro digestion method suitable for food—An international consensus. Food Funct..

[11] Rashid M.T., Liu K., Ning M., Jatoi M.A., Muzaffar N., Usman H. (2025). A gastronomic exploration of protein digestibility, antioxidant activity, and bioavailability of selenium-enriched germinated brown rice under various cooking methods. J. Agric. Food Res..

[12] Borugadda P., Kalluri H.K. (2025). A Comprehensive Analysis of Artificial Intelligence, Machine Learning, Deep Learning and Computer Vision in Food Science. J. Future Foods.

[13] Chen D., Guo C., Lu W., Zhang C., Xiao C. (2023). Rapid quantification of royal jelly quality by mid-infrared spectroscopy coupled with backpropagation neural network. Food Chem..

[14] Ma P., Li A., Yu N., Li Y., Bahadur R., Wang Q., Ahuja J.K. (2021). Application of machine learning for estimating label nutrients using USDA Global Branded Food Products Database, (BFPD). J. Food Compos. Anal..

[15] Kaur S., Singh N., Dagar P., Kumar A., Jaiswal S., Singh B.K., Bhardwaj R., Chand Rana J., Riar A. (2024). Comparative analysis of modified partial least squares regression and hybrid deep learning models for predicting protein content in Perilla (*Perilla frutescens* L.) seed meal using NIR spectroscopy. Food Biosci..

[16] Zhou M., Wang L., Wu H., Li Q., Li M., Zhang Z., Zhao Y., Lu Z., Zou Z. (2022). Machine learning modeling and prediction of peanut protein content based on spectral images and stoichiometry. LWT.

[17] Zhang X., Xu J., Sun Y., Zhang H., Guo S. (2025). Alkaline-heat induced the conformationally flexible regions of soy protein and their effect on subunit aggregation. Food Chem..

[18] (2016). National Food Safety Standard—Determination of Protein in Food.

[19] Zhang S., Yang L., Nie Y., Li H., Zhu D., Cao X., Fan H. (2025). Effects of thermal treatment and Glucono-δ-lactone on the quality of alkaline dough and steamed buns. Food Chem..

[20] Dong C., Zhao J., Jiang J. (2025). Cysteine-induced disulfide cleavage enhances the solubility of alkali-treated pea protein and its elasticity contribution in low-salt hybrid meat gels. Food Chem..

[21] Zhu N., Zang M., Wang S., Zhang S., Zhao B., Liu M., Li S., Wu Q., Liu B., Zhao Y. (2022). Modulating the structure of lamb myofibrillar protein gel influenced by psyllium husk powder at different NaCl concentrations: Effect of intermolecular interactions. Food Chem..

[22] Tanger C., Andlinger D.J., Brümmer-Rolf A., Engel J., Kulozik U. (2021). Quantification of protein-protein interactions in highly denatured whey and potato protein gels. MethodsX.

[23] Yin J., Liu X., Hu Z., Zhao H., Li C., Wang L. (2025). Effects of ultrasound-assisted alkaline isoelectric precipitation on the structure and functionality of Auricularia delicata protein. Innov. Food Sci. Emerg..

[24] Cao X., Liu H., Yang M., Mao K., Wang X., Chen Z., Ran M., Hao L. (2025). Evaluation of the nutritional quality of yeast protein in comparison to animal and plant proteins using growing rats and INFOGEST model. Food Chem..

[25] Dupont D., Mandalari G., Molle D., Jardin J., Léonil J., Faulks R.M., Wickham M.S., Mills E.N., Mackie A.R. (2010). Comparative resistance of food proteins to adult and infant in vitro digestion models. Mol. Nutr. Food Res..

[26] Ma C., Xia S., Song J., Hou Y., Hao T., Shen S., Li K., Xue C., Jiang X. (2024). Yeast protein as a novel protein source: Processing, functional properties, and potential applications in foods. Innov. Food Sci. Emerg..

[27] Liao L., Wang Q., Zhao M.-m. (2013). Functional, conformational and topographical changes of succinic acid deamidated wheat gluten upon freeze- and spray-drying: A comparative study. LWT—Food Sci. Technol..

[28] Oliete B., Yassine S.A., Cases E., Saurel R. (2019). Drying method determines the structure and the solubility of microfluidized pea globulin aggregates. Food Res. Int..

[29] Lam R.S.H., Nickerson M.T. (2013). Food proteins: A review on their emulsifying properties using a structure–function approach. Food Chem..

[30] Klassen D.R., Nickerson M.T. (2012). Effect of pH on the formation of electrostatic complexes within admixtures of partially purified pea proteins (legumin and vicilin) and gum Arabic polysaccharides. Food Res. Int..

[31] Xu L., Wang Y., Yang Y., Qiu C., Jiao A., Jin Z. (2024). Pea protein/carboxymethyl cellulose complexes prepared using a pH cycle strategy as stabilizers of high internal phase emulsions for 3D printing. Int. J. Biol. Macromol..

[32] Silva-Espinoza M.A., Ayed C., Foster T., Camacho M.D.M., Martínez-Navarrete N. (2019). The Impact of Freeze-Drying Conditions on the Physico-Chemical Properties and Bioactive Compounds of a Freeze-Dried Orange Puree. Foods.

[33] Jaeger A., Ahern N., Sahin A.W., Nyhan L., Mes J.J., van der Aa C., Vrasidas I., Arendt E.K. (2024). Dynamic in-vitro system indicates good digestibility characteristics for novel upcycled plant protein; correlation to techno-functional properties. Innov. Food Sci. Emerg..

[34] Li S., Mao X., Diao X., Yang K., Shan K., Li C. (2024). Effects of sodium tripolyphosphate on the quality and digestion properties of PSE pork. Food Chem..

[35] Yang J., Zhao W., Yu Y., Ren Y., Qian J.-Y. (2025). Insights into transglutaminase on structural and rheological properties of gels from adzuki bean protein pretreated by electric fields. Food Hydrocoll..

[36] Benrezkallah D. (2024). Molecular dynamics simulations at high temperatures of the *Aeropyrum pernix* L7Ae thermostable protein: Insight into the unfolding pathway. J. Mol. Graph. Model..

[37] Wang Y., Chen X., Xu X., Du M., Wu C. (2023). Reducing disulfide bonds as a robust strategy to facilitate the self-assembly of cod protein fabricating potential active ingredients-nanocarrier. Colloids Surf. B Biointerfaces.

[38] Giri S.K., Mangaraj S. (2012). Processing Influences on Composition and Quality Attributes of Soymilk and its Powder. Food Eng. Rev..

[39] Guzzo A., Ranganathan S., Gribskov M., Nakai K., Schönbach C. (2019). Data Storage and Representation. Encyclopedia of Bioinformatics and Computational Biology.

[40] Zhang L., Yang S., Wang C., Jiang Q., Wang X., Sun B. (2025). Moderately mechanically activated starch in improving protein digestibility: Application in noodles. Int. J. Biol. Macromol..

[41] Carvajal-Mena N., Tabilo-Munizaga G., Pérez-Won M., Herrera-Lavados C., Moreno-Osorio L. (2024). Influence of starch-protein interactions on the digestibility and chemical properties of a 3D-printed food matrix based on salmon by-product proteins. Food Res. Int..

[42] Szatko M., Konefał R., Njoku S., Zwoliński K., Andruniów T., Szweda R. (2025). Solvent effect on secondary structures of discrete, isotactic, oligourethane motif–towards engineering protein-like features in abiotic polymers. Eur. Polym. J..

[43] Rieder A., Afseth N.K., Böcker U., Knutsen S.H., Kirkhus B., Mæhre H.K., Ballance S., Wubshet S.G. (2021). Improved estimation of in vitro protein digestibility of different foods using size exclusion chromatography. Food Chem..

[44] García-Valle D.E., López-Silva M., Santos-Martínez G., Hernández-Pérez V., Figueroa-González J.J. (2024). Chemical, structural characterization and in vitro protein digestibility of cicada (*Cicadidae*) flour. J. Food Compos. Anal..

[45] Colombo R., Frosi I., Papetti A., Martínez-Villaluenga C., Hernández-Ledesma B. (2024). Chapter 1–Food protein digestion by in vitro static approaches. Protein Digestion-Derived Peptides.

[46] Khan Z.A., Hussain T., Ullah A., Ullah W., Del Ser J., Muhammad K., Sajjad M., Baik S.W. (2023). Modelling Electricity Consumption During the COVID19 Pandemic: Datasets, Models, Results and a Research Agenda. Energy Build..

[47] Li H., Wang Y., Zhang J., Li X., Wang J., Yi S., Zhu W., Xu Y., Li J. (2023). Prediction of the freshness of horse mackerel (*Trachurus japonicus*) using E-nose, E-tongue, and colorimeter based on biochemical indexes analyzed during frozen storage of whole fish. Food Chem..

[48] Zhang R., Zhong Y., Wang D., Gong L., Yang L., Guo F., Zhou G., Deng Y. (2025). Generative adversarial network integrated with metabolomics identifies potential biomarkers related to quality changes of atemoya (*Annona cherimola* × *Annona squamosa*) stored at 10 and 25 °C. Food Chem..

[49] Huang Q., Qiao C., Jing K., Zhu X., Ren K. (2022). Biomarkers identification for Schizophrenia via VAE and GSDAE-based data augmentation. Comput. Biol. Med..

[50] Gbashi S., Maselesele T.L., Njobeh P.B., Molelekoa T.B.J., Oyeyinka S.A., Makhuvele R., Adebo O.A. (2023). Application of a generative adversarial network for multi-featured fermentation data synthesis and artificial neural network (ANN) modeling of bitter gourd–grape beverage production. Sci. Rep..

[51] Huang X., You Y., Zeng X., Liu Q., Dong H., Qian M., Xiao S., Yu L., Hu X. (2024). Back propagation artificial neural network (BP-ANN) for prediction of the quality of gamma-irradiated smoked bacon. Food Chem..

[52] Fu B., Lei H., Ullah I., El-Meligy M., El Hindi K., Javed M.F., Ahmad F. (2025). Predictive modeling for durability characteristics of blended cement concrete utilizing machine learning algorithms. Case Stud. Constr. Mater..

[53] Zaher M., Ghoneim A.S., Abdelhamid L., Atia A. (2025). Fusing CNNs and attention-mechanisms to improve real-time indoor Human Activity Recognition for classifying home-based physical rehabilitation exercises. Comput. Biol. Med..

[54] Lu H., Song A., Li M., Yao X., Cai Y., Dong L., Kang D., Liu Y. (2025). Evaluation of the freshness (TVB-N) of pork patty during storage based on PLS-DA, SVM and BP-ANN models. Food Control.

[55] Chowdhury M.Z.I., Leung A.A., Walker R.L., Sikdar K.C., O’Beirne M., Quan H., Turin T.C. (2023). A comparison of machine learning algorithms and traditional regression-based statistical modeling for predicting hypertension incidence in a Canadian population. Sci. Rep..

[56] Sun K., Lan T., Goh Y.M., Safiena S., Huang Y.-H., Lytle B., He Y. (2024). An interpretable clustering approach to safety climate analysis: Examining driver group distinctions. Accid. Anal. Prev..

[57] Alrosan M., Madi Almajwal A., Al-Qaisi A., Gammoh S., Alu’datt M.H., Al Qudsi F.R., Tan T.-C., Razzak Mahmood A.A., Bani-Melhem K. (2024). Trehalose-conjugated lentil-casein protein complexes prepared by structural interaction: Effects on water solubility and protein digestibility. Food Chem..

[58] Alrosan M., Almajwal A.M., Al-Qaisi A., Gammoh S., Alu’datt M.H., Al Qudsi F.R., Tan T.-C., Razzak Mahmood A.A., Maghaydah S., Al-Massad M. (2024). Evaluation of digestibility, solubility, and surface properties of trehalose-conjugated quinoa proteins prepared via pH shifting technique. Food Chem. X.

[59] Hegde K.R., Buvaneswaran M., Bhavana M.R., Sinija V.R., Rawson A., Hema V. (2025). Effects of ultrasound and high-pressure assisted extraction of pearl millet protein isolate: Functional, digestibility, and structural properties. Int. J. Biol. Macromol..

[60] Wang J., Wang J., Kranthi Vanga S., Raghavan V. (2021). Influence of high-intensity ultrasound on the IgE binding capacity of Act d 2 allergen, secondary structure, and In-vitro digestibility of kiwifruit proteins. Ultrason. Sonochem..

[61] Qin N., Shuang Q., Bao X. (2025). High-pressure processing-assisted limited enzymatic hydrolysis improves the luminosity, functional properties, and digestibility of sunflower meal protein. LWT.

